# Smoking Exposure Is Associated with Serum Vitamin D Deficiency in Children: Evidence from the Japan Environment and Children’s Study

**DOI:** 10.3390/nu14153121

**Published:** 2022-07-29

**Authors:** Limin Yang, Miori Sato, Mayako Saito-Abe, Yumiko Miyaji, Chikako Sato, Minaho Nishizato, Natsuhiko Kumasaka, Hidetoshi Mezawa, Kiwako Yamamoto-Hanada, Yukihiro Ohya

**Affiliations:** 1 Allergy Center, National Center for Child Health and Development, Tokyo 157-8535, Japan; sato-m@ncchd.go.jp (M.S.); saito-myk@ncchd.go.jp (M.S.-A.); miyaji-y@ncchd.go.jp (Y.M.); sato-ch@ncchd.go.jp (C.S.); nishizato-m@ncchd.go.jp (M.N.); kumasaka-n@ncchd.go.jp (N.K.); mezawa-h@ncchd.go.jp (H.M.); yamamoto-k@ncchd.go.jp (K.Y.-H.); ohya-y@ncchd.go.jp (Y.O.); 2 Medical Support Center for the Japan Environment and Children’s Study, National Research Institute for Child Health and Development, Tokyo 157-8535, Japan

**Keywords:** tobacco smoke exposure, children, serum 25(OH)D, vitamin D, deficiency

## Abstract

Tobacco smoke exposure is known to lower serum 25-hydroxyvitamin D (25(OH)D) concentrations. This study evaluated the association between passive smoking and vitamin D deficiency (VDD) in young children using data from the Japan Environment and Children’s Study (JECS), the largest birth cohort study in Japan. Information on parental smoking status was extracted from a survey of JECS for children aged 1.5 years and data for serum 25(OH)D concentrations were obtained from blood tests in the Sub-Cohort Study of JECS performed at age 2 years. Logistic regression and linear models were fitted to evaluate the association between these variables. Data were analyzed for 4593 children. After adjusting for covariates, smoke exposure was significantly associated with increased incidence of VDD (OR 1.35; 95% CI, 1.14–1.59) according to the logistic model. The linear model indicated that passive smoking negatively predicted de-seasonalized serum 25(OH)D concentrations (β −0.5; 95% CI −0.95 to −0.08) in children aged 2 years. The results suggest that smoke exposure is a risk factor for VDD in children. Given that VD plays a crucial role in bone metabolism and the immune system, our findings are significant for clinical and public health.

## 1. Introduction

Vitamin D (VD) is a steroid hormone that can be obtained from exposure to sunshine and by dietary intake. The two forms of VD are ergocalciferol (VD2) and cholecalciferol (VD3). From exposure to sunlight, specifically ultraviolet B radiation, previtamin D3 is synthesized from 7-dehydroxycholesterol through photolytic conversion [1], and vitamin D3 is converted from previtamin D3 in the skin. In the liver, 25-hydroxyvitamin D (25(OH)D)—the major circulating form and the best producerr of VD—is generated by the action of 25-hydroxylase. Finally, 25(OH)D is metabolized in the kidney to 1,25-dihydroxyvitamin D3 [1], a biologically active metabolite of VD [2,3].

Vitamin D plays a crucial role in regulating calcium and phosphate metabolism and maintaining bone density [4], whereas its deficiency is suggested to be associated with osteoporosis, fractures, and rickets in the young population [5]. In addition to bone health, VD has anti-inflammatory and antioxidant properties and affects the immune system by regulating immune processes [1]. Vitamin D deficiency (VDD) appears to be related to chronic disorders, such as cancers, diabetes, autoimmune diseases, and heart diseases [6]. According to an epidemiologic study, VDD risk factors include lack of sun exposure, physical inactivity, obesity, and measurements in the winter season [6]. Globally, more than 1 billion people have VDD [7].

Cigarettes and cigarette smoke contain more than 7000 chemicals, and most of these chemicals are harmful, such as nicotine, hydrogen cyanide, formaldehyde, and lead, [8]. Tobacco smoke exposure is considered to lower serum 25(OH)D concentrations, and then lead to low bone mass or fracture [9]. Although the results remain inconsistent, this depressed effect has been reported among different adult populations. Studies have also been performed in children [9,10,11]. An analysis for young population (3–17 years US children) indicated that tobacco smoke exposure is associated with an increased risk of vitamin D deficiency in young population [11]. However, little is known about the Japanese population, especially in young children. Therefore, we designed this study to evaluate the association between passive smoking and VDD in young children using data from a birth cohort in Japan.

## 2. Materials and Methods

### 2.1. Study Design

The participants in this observational study were obtained from the Japan Environment and Children’s Study (JECS), the largest birth cohort study in Japan [12]. The main aims of JECS were to evaluate the association between exposure to environmental chemicals and children’s health and development [12]. The recruitment period for JECS was between January 2011 and March 2014 from 15 Regional Centers [12] The parents and their children were followed until children reached 13 years of age [12].

Data collection methods included self-administered questionnaires, environmental measurements, developmental and neuropsychological tests, and medical checks according to the JECS protocols [12]. Medical checks, including blood tests, were performed at 2, 4, 6, 8, 10, and 12 years as a Sub-Cohort Study of JECS [12,13].

**Figure 1:**
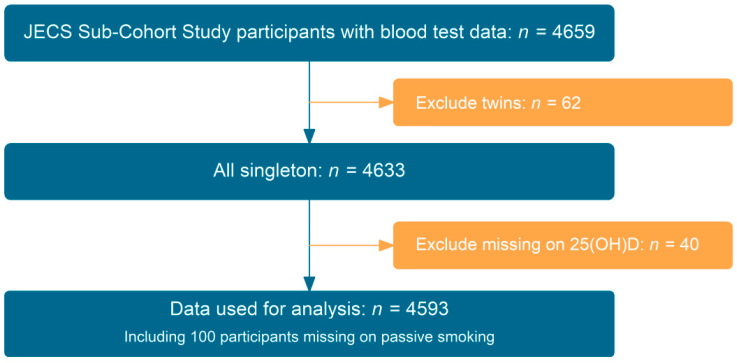
Study flow-chart. JECS, Japan Environment and Children’s Study.

Our study was designed to explore children’s exposure to tobacco smoke in relation to VDD. Information on parental smoking status was extracted from the self-administered questionnaire conducted when children were age 1.5 years, and data for serum 25(OH)D concentrations were obtained from blood tests in the Sub-Cohort Study of JECS performed at age 2 years. After excluding children with missing 25(OH)D data, we analyzed 4593 children in this study. Detailed data selection is presented in Figure 1. The datasets jecs-ta-20190930 and jecs-ta-20190930-mdv, released by the Program Office in October 2019, were used for analysis.

### 2.2. Assessment of Children’s Tobacco Smoke Exposure

Passive smoking was defined by the answer “Yes” to the question “Do you smoke currently?” as answered by the mother or father. Another question that addressed the number of cigarettes smoked per day at home was used to assess the dose–response relationship between tobacco smoke exposure and VDD. The number of cigarettes smoked per day was grouped as a category variable (0, 1–5, 6–10 and ≥11).

### 2.3. Model Covariates

The covariates were determined based on scientific knowledge and published studies on the determinants of 25(OH)D. Sociodemographic data included education level [11] and annual household income [11], which were asked on the MT2 questionnaire [14]. Children’s sex [11], gestational age at birth [15,16], and birthweight [11,17] were extracted from the medical records after delivery [14]. Other model covariates, including daycare attendance, wearing a hat playing outside, outside play time (associated with sunlight exposure) and Z scores of body mass index (BMI) [11] at age 1.5 years, were obtained from the questionnaire conducted after birth. The timing (the month) of the blood test [6] was extracted from the Sub-Cohort Study of JECS at 2 years and grouped into 4 seasons of 3 months each.

### 2.4. Serum 25(OH)D Concentrations

Serum 25(OH)D concentration was measured by the liquid chromatography–tandem mass spectrometry (LC-MS/MS) method (LSI Medience Corporation, Tokyo, Japan) [18]. The intra-assay and inter-assay CV are described in our previous published study [18]. Serum 25(OH)D concentrations were defined as a sum of 25(OH)D2 and 25(OH)D3. Serum 25(OH)D concentrations less than 20 ng/mL were defined as VDD based on criteria published by the Japanese Society for Bone and Mineral Research [18].

### 2.5. Statistical Analysis

To assess the relationship between tobacco smoke exposure and VDD, we fitted the logistic regression model. We first fitted Model 1 with tobacco smoke exposure and children’s sex as the independent variables, and then we added sociodemographic factors, daycare attendance, and season of blood test into Model 2 (main model). Finally, the Z score of BMI at age 1.5 years was further adjusted.

We did not perform a stepwise selection to reduce variables in the models. The multicollinearity of the independent variables in the models was scrutinized for variance inflation factor values. We assumed that the missing data in the model were random, and missing data for covariates were imputed using multiple imputation (MI) analysis. The MI process generated 20 datasets with the missing data imputed. All confounders were used in the imputation processes.

Next, we explored the dose–response relationship of tobacco smoke exposure with VDD using the number of cigarettes per day as a proxy for magnitude of tobacco smoke exposure in children. In addition, we fitted the linear regression model for de-seasonalized serum 25(OH)D concentrations [19]. Calculation details for de-seasonalized serum 25(OH)D concentrations were published in our previous study [20]

As a sensitivity analysis, we also fitted the models with a complete dataset by removing data in the dataset if it contains missing values.

Descriptive analysis and modeling were conducted with R version 4.0.3 software (Institute for Statistics and Mathematics, Vienna, Austria; www.r-project.org accessed on 10 December 2020).

## 3. Results

### 3.1. Baseline Characteristic, Passive Smoking, and VDD

Figure 1 presents the flow chart for the study. After excluding children with missing data for serum 25(OH)D2 or 25(OH)D3 concentrations, 4593 children remained for analysis. Table 1 presents the baseline characteristics of the study cohort. Among the 4493 children whose parents answered the question involving smoking, 1289 (28.7%) children were judged to be exposed to tobacco smoke. The prevalence of VDD was near 25% in this cohort. Tables S1 and S2 show the baseline characteristics for children by VDD or passive smoking.

### 3.2. Association between Passive Smoking at Age 1.5 Years and VDD at 2 Years

Table 2 lists the results from logistic regression models, which identified the association between passive smoking in children and VDD. After adjusting for children’s sex, parents’ education levels, income, low birth weight, premature birth, ages of mother at pregnancy, exclusive breast milk before and at 6 months, day nursery, wearing a hat playing outside, outside play time and season of blood test, passive smoking in children was significantly associated with an increased incidence of VDD at 2 years (OR 1.35; 95% CI, 1.14–1.59). The inclusion of BMI z-score in logistic models did not meaningfully change the relationship between smoke exposure and VDD.

In addition, there does not appear to be a dose–response relationship between the number of cigarettes smoked indoors and VDD. Compared with children who had no second-hand smoke exposure at home, after covariate adjustment, only children whose fathers smoked 1–5 cigarettes per day indoors was associated with an increased incidence of VDD at age 2 years (OR 1.47; 95% CI, 1.17–1.85 for fathers smoking 1–5 cigarettes/day; OR 1.10; 95% CI, 0.82–1.48 for 6–10 cigarettes/day; OR 1.25; 95% CI, 0.89–1.77 for 11 or more cigarettes/day). A similar pattern was also observed in models fitted with the complete dataset (Table S3).

### 3.3. Association between Passive Smoking at Age 1.5 Years and De-Seasonalized Serum 25(OH)D Concentrations at Age 2 Years

Table 3 shows the association between passive smoking and de-seasonalized serum 25(OH)D concentrations. As shown, passive smoking negatively predicted de-seasonalized serum 25(OH)D concentrations in the models. After adjusting for the children’s sex, parents’ education levels, income, low birth weight, premature birth, ages of mother at pregnancy, exclusive breast milk before and at 6 months, day nursery, wearing a hat playing outside and outside play time, compared with the non-exposure group, passive smoking was associated with low de-seasonalized serum 25(OH)D concentrations (β −0.5; 95% CI, −0.95 to −0.08). Passive smoking was still significantly negatively associated with de-seasonalized 25(OH)D, and the coefficient was almost unchanged (Model 3 in Table 3), when the Z scores of BMI at age 1.5 years were added into the model. The linear model for serum 25(OH)D concentrations showed similar results to those for de-seasonalized serum 25(OH)D concentrations (data not shown). The results for the relationship between the number of cigarettes smoked per day by parents and VDD in children showed that only the group smoking 1–5 cigarettes per day was significantly associated with decreased de-seasonalized serum 25(OH)D concentrations, compared with the non-exposure group (β −1.17; 95% CI, −2.33 to −0.01 for mothers smoking 1–5 cigarettes/day; β −0.74; 95% CI, −1.36 to −0.12 for fathers smoking 1–5 cigarettes/day).

## 4. Discussion

Our results showed a high risk of VDD in children exposed to parental smoking compared with children who were not exposed. This study is the first effort to assess the association between smoking and VDD in children as young as 2 years in the Japanese population on this topic, to our best knowledge.

Our results are in line with those of several published studies. A recent report from the U.K. Biobank indicated that tobacco smoking increased the risk of VDD in adults [21]. Similarly, a study in Finland among 5714 adults age 30–79 years showed lower serum 25(OH)D concentrations in former and current smokers than never- or less than 1-year smokers [6]. Evidence from a study in Sweden in young men showed that smokers consistently had lower serum VD concentrations than nonsmokers [22]. In addition, significantly low serum 25(OH)D3 levels were observed in both active and passive smoking groups, according to the report from Soldin et al. who evaluated steroid hormone concentrations of 293 women age 18–45 years in the United States [23]. A study in Chinese men found an inverse dose–response relationship—the greater the number of cigarettes per day, the lower the concentration of VD [24]. Maternal smoke exposure also has been found to be associated with low serum VD in infants [15]. Moreover, Chinellato et al. evaluated the effect of passive smoking exposure on serum VD concentrations in 152 white children and found significantly lower serum 25(OH)D concentrations among those with exposure to both parents smoking than those without smoking exposure [10]. In contrast, a study in Iran by Banihosseini et al. did not find a significant association [25]; however, the lack of power due to a limited sample size may explain the nonsignificant effect of smoking on VD in the study.

The mechanisms for lower VD with smoking exposure remain unclear. Some molecular pathways have been reported. Smoking exposure causes a disruption of VD metabolism, related to the dysfunction of VD-parathyroid hormone (PTH) axis [5]. The simultaneous decrease in PTH and calcitriol has been observed in a population with smoking exposure [26]. The reduction in 1α-hydroxylase with exposure to cigarette smoking extract also has been reported. In addition, the dysregulation of genes that encode for the enzymes involving VD metabolism may explain the deleterious effect of smoking exposure on serum VD [5]. The decreased concentration of serum VD with smoking exposure may also be related to disturbances in food intake. A study evaluating smoking exposure, dietary calcium, and VD concentrations in women showed that tobacco smoking can change the taste of dietary intake, and thereby result in lower intake of VD from food; moreover, this change can be reversed by smoking cessation [5,27].

We did not find a dose–response relationship between the number of cigarettes smoked indoors and VDD. The number of cigarettes smoked by parents may not a good proxy to reflect the true magnitude of tobacco smoke exposure in children. The overall tobacco smoke exposure from children’s environments included second-hand smoke and third-hand smoke, which is residual nicotine and other smoke toxicants accumulated on dust, the surface of clothes and furniture in children’s environments by smokers. Therefore, the tobacco smoke exposure was affected not only by number of cigarettes smoked per day, but also by other determinants, such as the number of indoor smokers, the size of smoking space, the ventilation frequency, efficiency of the ventilation, and children’s indoor time. A further evaluation of the effects of smoking on VDD should focus on tobacco smoke exposure with better biomarkers, such as hand nicotine or salivary/urinary cotinine.

The large sample size is an important strength of our study. The data used for analysis were obtained from the largest birth cohort in Japan, which could provide enough power to show the significant association between smoking exposure and VDD in the model. Moreover, LM-MS/MS was used to test serum 25(OH)D concentrations. This method is considered to be a reliable and robust method of measurement, and is extensively used in studies involving serum 25(OH)D concentrations as a gold standard [28,29]. In addition, although the study covers a wide geographic area from the northernmost prefecture (Hokkaido) to the southernmost prefecture (Okinawa); all tests for serum 25(OH)D concentrations were performed in one laboratory, which minimized the potential measurement errors.

Comparison with other studies should be performed with caution. Although serum 25(OH)D concentration is the best marker to reflect VD concentrations in the body, no single cutoff value has been agreed upon by health agencies for serum 25(OH)D concentration to define VDD. Second, serum VD status was evaluated according to a single blood test, which may not reflect long-term VD in the body very well [20,21]. Third, a value of 25(OH)D was truncated as 4 in this study if a value of serum 25(OH)D2 or 25(OH)D3 was lower than 4 ng/mL, which may cause an overestimation of VD level [18,20]. Fourth, the information on tobacco smoke exposure was collected from the questionnaires, which may underreport smoking and could not reflect the precise degree of exposure to tobacco overall. Finally, although the analysis was performed on data from a large cohort, we cannot obtain a causal conclusion because of the observational study design. The results warrant replications in other young populations to confirm our findings.

In conclusion, our data suggest that children’s tobacco smoke exposure is a risk factor for VDD. Given that VD plays crucial role in the bone metabolism and the immune system, our findings have important clinical and public health significance. Further studies are needed to clarify the genetic mechanisms of this relationship.

## Figures and Tables

**Table 1:** Baseline characteristics of participants.

Variables		N	%
Education level of mother	High	3158	69.1
	Low	1413	30.9
	Missing	22	
Education level of father	High	2774	60.9
	Low	1780	39.1
	Missing	39	
Annual Income of family	Normal or high	2777	63.3
	Low	1610	36.7
	Missing	206	
Low birth weight	No	4257	92.7
	Yes	336	7.3
Sex of children	Boys	2338	50.9
	Girls	2255	49.1
Premature birth	No	4415	96.1
	Yes	178	3.9
Ages of mother at pregnancy	<35	3250	70.8
	≥35	1342	29.2
	Missing	1	
Exclusive breast milk before and at 6 months	No	2795	61
	Yes	1787	39
	Missing	11	
Day nursery	No	2318	51.5
	Yes	2180	48.5
	Missing	95	
Season of 25(OH)D measurement	March–May	998	21.7
	June–August	1441	31.4
	September–November	1403	30.5
	December–February	751	16.4
Wearing a hat playing outside	Yes	3508	77.7
	No	1004	22.3
	Missing	81	
Outside play time (hours)	<1	2060	46.3
	≥1	2390	53.7
	Missing	143	
Parents smoking	No	3204	71.3
	Yes	1289	28.7
	Missing	100	
Number of cigarettes smoked at home (mother)	0	4229	93.7
	1–5	122	2.7
	6–10	103	2.3
	≥11	60	1.3
	Missing	79	
Number of cigarettes smoked at home (father)	0	3256	76.2
	1–5	500	11.7
	6–10	307	7.2
	≥11	208	4.9
	Missing	322	
Serum 25(OH)D concentrations (ng/mL)	≥20	3459	75.3
	<20	1134	24.7
Z scores of BMI at 18 months mean (SD)		0.48	(1.13)

**Table 2:** Association of tobacco smoke exposure with vitamin D deficiency in children age 2 years.

			95% CI		
		OR	Lower	Upper	*p* Value
Tabaco smoking exposure ^#^					
Model 1 ^a^		**1.34**	**1.16**	**1.55**	**0.0001**
Model 2 ^b^		**1.35**	**1.14**	**1.59**	**0.0004**
Model 3 ^c^		**1.34**	**1.14**	**1.59**	**0.0005**
Number of cigarettes smoked at home (mother) ^$^					
Model 1 ^a^					
	1–5	**1.59**	**1.08**	**2.33**	**0.018**
	6–10	0.79	0.49	1.28	0.340
	≥11	**1.72**	**1.00**	**2.94**	**0.049**
Model 2 ^b^					
	1–5	1.38	0.90	2.11	0.137
	6–10	0.72	0.43	1.23	0.228
	≥11	1.65	0.91	3.00	0.010
Model 3 ^c^					
	1–5	1.39	0.91	2.12	0.134
	6–10	0.74	0.43	1.25	0.253
	≥11	1.64	0.90	2.98	0.104
Number of cigarettes smoked at home (father) ^$^					
Model 1 ^a^					
	1–5	**1.51**	**1.23**	**1.85**	**<0.001**
	6–10	1.07	0.82	1.40	0.635
	≥11	1.22	0.89	1.66	0.225
Model 2 ^b^					
	1–5	**1.47**	**1.17**	**1.85**	**0.001**
	6–10	1.10	0.82	1.48	0.527
	≥11	1.25	0.89	1.77	0.202
Model 3 ^c^					
	1–5	**1.46**	**1.16**	**1.84**	**0.001**
	6–10	1.10	0.82	1.49	0.514
	≥11	1.25	0.89	1.77	0.201

Models were fitted with logistic regression model. OR odds ratios, CI; confidential inference; ^a^ Model 1 adjusted sex; ^b^ Model 2 adjusted sex, education levels of parents, income, low birth weight, premature birth, ages of mother at pregnancy, exclusive breast milk before and at 6 months, day nursery, wearing a hat playing outside, outside play time and season of blood test; ^c^ Model 3 adjusted sex, education levels of parents, income, low birth weight, premature birth, ages of mother at pregnancy, exclusive breast milk before and at 6 months, day nursery, wearing a hat playing outside, outside play time, season of blood test and z scores of BMI at age 1.5 years; ^#^ No smoking exposure was used as reference category; ^$^ Not smoking at home group was reference group.

**Table 3:** Association between tobacco smoke exposure and de-seasonalized serum 25(OH)D concentrations in children aged 2 years.

			95% CI		
		Coefficients	Lower	Upper	*p* Value
Tabaco smoking exposure					
Model 1 ^a^		**−0.55**	**−0.97**	**−0.13**	**0.011**
Model 2 ^b^		**−0.52**	**−0.95**	**−0.08**	**0.020**
Model 3 ^c^		**−0.51**	**−0.95**	**−0.08**	**0.021**
Number of cigarettes smoked at home (mother)					
Model 1 ^a^					
	1–5	**−1.21**	**−2.37**	**−0.05**	**0.041**
	6–10	0.53	−0.73	1.79	0.412
	≥11	0.50	−1.18	2.18	0.558
Model 2 ^b^					
	1–5	**−1.17**	**−2.33**	**−0.01**	**0.048**
	6–10	0.53	−0.73	1.80	0.409
	≥11	0.54	−1.15	2.23	0.529
Model 3 ^c^					
	1–5	**−1.18**	**−2.34**	**−0.02**	**0.047**
	6–10	0.52	−0.75	1.78	0.424
	≥11	0.56	−1.13	2.25	0.518
Number of cigarettes smoked at home (father)					
Model 1 ^a^					
	1–5	**−0.85**	**−1.46**	**−0.24**	**0.007**
	6–10	−0.35	−1.10	0.40	0.363
	≥11	0.30	−0.63	1.22	0.529
Model 2 ^b^					
	1–5	**−0.74**	**−1.36**	**−0.12**	**0.020**
	6–10	−0.33	−1.08	0.42	0.392
	≥11	0.22	−0.71	1.15	0.648
Model 3 ^c^					
	1–5	**−0.73**	**−1.35**	**−0.11**	**0.021**
	6–10	−0.33	−1.09	0.42	0.387
	≥11	0.22	−0.71	1.15	0.647

Models were fitted with linear regression model. CI, confidential interval; ^a^ Model 1 adjusted for sex; ^b^ Model 2 for adjusted sex, education levels of parents, income, low birth weight, premature birth, ages of mother at pregnancy, exclusive breast milk before and at 6 months, day nursery, wearing a hat playing outside and outside play time; ^c^ Model 3 for adjusted sex, education levels of parents, income, low birth weight, premature birth, ages of mother at pregnancy, exclusive breast milk before and at 6 months, day nursery, wearing a hat playing outside, outside play time and z scores of BMI at age 1.5 years.

## Data Availability

Data are unsuitable for public deposition due to ethical restrictions and the legal framework of Japan. It is prohibited by the Act on the Protection of Personal Information (Act No. 57 of 30 May 2003, amendment on 9 September 2015) to publicly deposit data containing personal information. Ethical Guidelines for Medical and Health Research Involving Human Subjects enforced by the Japan Ministry of Education, Culture, Sports, Science and Technology and the Ministry of Health, Labour and Welfare also restricts the open sharing of the epidemiologic data. All inquiries about access to data should be sent to: jecs-en@nies.go.jp. The person responsible for handling enquiries sent to this e-mail address is Dr Shoji F. Nakayama, JECS Programme Office, National Institute for Environmental Studies.

## Supplementary Materials

The following are available online at https://www.mdpi.com/article/10.3390/nu14153121/s1, Table S1: Baseline characteristics by vitamin D deficiency; Table S2: Baseline characteristics by smoking exposure; Table S3: Logistic models evaluating the association of tobacco smoke exposure with vitamin D deficiency in children aged 2 years using the complete dataset.
