# Supplementary material for: Smoking Exposure Is Associated with Serum Vitamin D Deficiency in Children: Evidence from the Japan Environment and Children’s Study
Source: Nutrients. 2022 Jul 29;14(15):3121. doi: 10.3390/nu14153121 (PMC9370804; doi:10.3390/nu14153121)
Supplement: Supplementary file 1 [file nutrients-14-03121-s001.zip › nutrients-1805394-supplementary.pdf]

Supplementary files

Table S1. Baseline characteristics by vitamin D deficiency

Table S2. Baseline characteristics by smoking exposure

Table S3. Logistic models evaluating the association of tobacco smoke exposure with vitamin D deficiency in children age 2 years using the complete dataset

Table S1. Baseline characteristics by vitamin D deficiency.

Variables		Non-VDD		VDD	
		N	%	N	%
Education level of mother	High	2404	69.8	754	66.8
	Low	1039	30.2	374	33.2
Education level of father	High	2118	61.7	656	58.4
	Low	1312	38.3	468	41.6
Annual Income of family	Normal or high	2120	64.1	657	60.8
	Low	1187	35.9	423	39.2
Low birth weight	No	3214	92.9	1043	92
	Yes	245	7.1	91	8
Sex of children	Boys	1801	52.1	537	47.4
	Girls	1658	47.9	597	52.6
Premature birth	No	3334	96.4	1081	95.3
	Yes	125	3.6	53	4.7
Ages of mother at pregnancy	<35	2423	70.1	827	72.9
	>=35	1035	29.9	307	27.1
Exclusive breast milk	No	2156	62.5	639	56.5
before and at 6 months	Yes	1295	37.5	492	43.5
Day nursery	No	1678	49.6	640	57.5
	Yes	1707	50.4	473	42.5
Seasons of 25(OH)D measurement	March–May	623	18	375	33.1
	June–August	1266	36.6	175	15.4
	September–November	1214	35.1	189	16.7
	December–February	356	10.3	395	34.8
Wearing a hat playing outside	Yes	2650	78	858	77.1
	No	749	22	255	22.9
Outside play time (hours)	<1	1412	42	648	59.3
	>=1	1946	58	444	40.7
Parent smoking	No	2464	72.9	740	66.5
	Yes	917	27.1	372	33.5

Z scores of BMI at 18 months	Mean (SD)	0.52 (1.12)		0.37 (1.17)	

VDD: Vitamin D deficiency.

Table S2. Baseline characteristics by smoking exposure

Variables		No smoking exposure		Passive smoking	
		N	%	N	%
Education level of mother	High	2390	75	713	55.5
	Low	797	25	571	44.5
Education level of father	High	2151	67.7	577	45.1
	Low	1028	32.3	701	54.9
Annual Income of family	Normal or high	2113	68.7	615	50.5
	Low	962	31.3	604	49.5
Low birth weight	No	2973	92.8	1189	92.2
	Yes	231	7.2	100	7.8
Sex of children	Boys	1647	51.4	636	49.3
	Girls	1557	48.6	653	50.7
Premature birth	No	3077	96	1241	96.3
	Yes	127	4	48	3.7
Ages of mother at pregnancy	<35	2202	68.7	970	75.3
	>=35	1001	31.3	319	24.7
Exclusive breast milk	No	1909	59.7	833	64.8
before and at 6 months	Yes	1288	40.3	453	35.2
Day nursery	No	1657	52.4	628	50
	Yes	1507	47.6	627	50
Seasons of 25(OH)D measurement	March–May	681	21.3	292	22.7
	June–August	1018	31.8	386	29.9
	September–November	989	30.9	393	30.5
	December–February	516	16.1	218	16.9
Wearing a hat playing outside	Yes	2486	78.3	954	75.8
	No	689	21.7	304	24.2

Outside play time (hours)	<1	1450	46.3	574	46.4
	>=1	1684	53.7	664	53.6
Z scores of BMI at 18 months	Mean (SD)	0.50 (1.13)		0.45 (1.14)	

Table S3. Logistic models evaluating the association of tobacco smoke exposure with vitamin D deficiency in children age 2 years using the complete dataset

			95% CI		
		OR	Lower	Upper	p value
Tabaco smoking exposure[#]					
Model 1 [a]		1.29	1.09	1.52	0.003
Model 2 [b]		1.33	1.10	1.61	0.003
Model 3 [c]		1.32	1.09	1.60	0.004
Number of cigarettes smoked at home (mother) [$]					
Model 1 [a]					
	1–5	1.30	0.81	2.09	0.274
	6–10	0.76	0.42	1.36	0.351
	>=11	1.13	0.55	2.34	0.744
Model 2 [b]					
	1–5	1.15	0.68	1.94	0.613
	6–10	0.68	0.36	1.30	0.242
	>=11	1.14	0.51	2.54	0.755
Model 3 [c]					
	1–5	1.15	0.68	1.95	0.607
	6–10	0.70	0.37	1.33	0.273
	>=11	1.13	0.51	2.52	0.765
Number of cigarettes smoked at home (father) [$]					
Model 1 [a]					
	1–5	1.57	1.25	1.98	<0.001
	6–10	0.99	0.72	1.36	0.965
	>=11	1.17	0.81	1.69	0.403
Model 2 [b]					
	1–5	1.62	1.25	2.10	<0.001
	6–10	1.02	0.72	1.44	0.930
	>=11	1.18	0.78	1.77	0.429
Model 3 [c]					
	1–5	1.60	1.23	2.08	<0.001
	6–10	1.02	0.72	1.45	0.892
	>=11	1.18	0.78	1.77	0.430

Models were fitted with logistic regression model. OR odds ratios, CI; confidential inference; [a] Model 1 adjusted sex; [b] Model 2 adjusted sex, education levels of parents, income, low birth weight, premature birth, ages of mother at pregnancy, exclusive breast milk before and at 6 months, day nursery, wearing a hat playing outside, outside play time and season of blood test; [c] Model 3 adjusted sex, education levels of parents, income, low birth weight, premature birth, ages of mother at pregnancy, exclusive breast milk before and at 6 months, day nursery, wearing a hat playing outside, outside play time, season of blood test and z scores of BMI at age 1.5 years; [#]No smoking exposure was used as reference category; [$]Not smoking at home group was reference group.
